# Combined Strategy Using High Hydrostatic Pressure, Temperature and Enzymatic Hydrolysis for Development of Fibre-Rich Ingredients from Oat and Wheat By-Products

**DOI:** 10.3390/foods13030378

**Published:** 2024-01-24

**Authors:** Iván Jesús Jiménez-Pulido, Daniel Rico, Daniel De Luis, Ana Belén Martín-Diana

**Affiliations:** 1 Agrarian Technological Institute of Castilla and Leon (ITACyL), Ctra. Burgos Km 119, Finca Zamadueñas, 47071 Valladolid, Spain; jimpuliv@itacyl.es (I.J.J.-P.); mardiaan@itacyl.es (A.B.M.-D.); 2 Endocrinology and Nutrition Research Centre, Medicine School, Service of Endocrinology and Nutrition, Universitary Clinic Hospital of Valladolid, University of Valladolid, Av. Ramón y Cajal, 3, 47003 Valladolid, Spain; dluisro@saludcastillayleon.es

**Keywords:** high pressure thermal (HPP), ultraFloXL, viscoferm, oat, wheat, bran, fibre hull, antioxidant, β-glucans, phytic acid

## Abstract

Wheat bran (WB) and oat hull (OH) are two interesting undervalued cereal processing sources rich in total dietary fibre (TDF) and other associated bioactive compounds, such as β-glucans and polyphenols. The aim of this study was to optimise a combination chemical (enzymes) and physical (high hydrostatic pressure-temperature) strategies to increase the bioaccessibility of bioactive compounds naturally bound to the bran and hull outer layers. WB and OH were hydrolysed using food-grade enzymes (UltraFloXL and Viscoferm, for WB and OH, respectively) in combination with HPP at different temperatures (40, 50, 60 and 70 °C) and hydrolysis either before or after HPP. Proximal composition, phytic acid, β-glucans, total phenolics (TPs) and total antioxidant activity (TAC) were evaluated to select the processing conditions for optimal nutritional and bioactive properties of the final ingredients. The application of the hydrolysis step after the HPP treatment resulted in lower phytic acid levels in both matrices (WB and OH). On the other hand, the release of β-glucan was more effective at the highest temperature (70 °C) used during pressurisation. After the treatment, the TP content ranged from 756.47 to 1395.27 µmol GAE 100 g^−1^ in WB, and OH showed values from 566.91 to 930.45 µmol GAE 100 g^−1^. An interaction effect between the temperature and hydrolysis timing (applied before or after HPP) was observed in the case of OH. Hydrolysis applied before HPP was more efficient in releasing OH TPs at lower HPP temperatures (40–50 °C); meanwhile, at higher HPP temperatures (60–70 °C), hydrolysis yielded higher TP values when applied after HPP. This effect was not observed in WB, where the hydrolysis was more effective before HPP. The TP results were significantly correlated with the TAC values. The results showed that the application of optimal process conditions (hydrolysis before HPP at 60 or 70 °C for WB; hydrolysis after HPP at 70 °C for OH) can increase the biological value of the final ingredients obtained.

## 1. Introduction

The relationship between food and health is complex and multifaceted. A balanced and nutritious diet is essential for maintaining good health, preventing diseases and promoting overall well-being. The interest of consumers in including fibre in their diet has experienced an exponential growth, mostly driven by a better understanding of the health benefits associated with fibre consumption. The WHO/FAO and EFSA recommend a fibre intake of 18–38 g/day for adults, and to ensure this daily amount in the diet, it is necessary to consume fruits, vegetables and cereals [1,2,3,4].

The processing of cereals generates byproducts such as hull and bran; the properties of these outer coats depend largely on the type of grain, size, shape, germ size, pericarp thickness and the milling process used [5]. WB contains 37–53% DF, of which 95% is an insoluble fraction [6]. OH makes up 25–35% of the entire grain and is mainly composed of insoluble fibre (lignin, cellulose and hemicellulose) and β-glucans [7,8].

WB and OH consumption are associated with health benefits that have been reported by many authors [9,10]. The European Food Safety Authority regards both as beneficial to health [11]. 

Fibre is recognised for its role in promoting digestive health, managing weight, and reducing the risk of chronic diseases, often associated with its effect on the regulation of bowel function [12]. The rising prevalence of type II diabetes also affects dietary choices by consumers in favour of fibre-rich food, especially in the case of soluble fibre, which can help regulate glucose blood levels and slow down its absorption [13]. Cellulose and hemicellulose structures from wheat bran and oat hull are rich in phenolic acids (ferulic acid, p-coumaric acid, dihydroxybenzoic acid and sinapic acid) and other interesting bioactive compounds in the case of oat, such as avenanthramides [14,15]. The bound nature of these phenolic compounds makes the conventional extraction procedures inefficient. Furthermore, under certain extraction conditions (excessive light and air/oxygen exposure and alkaline hydrolysis), the stability of the phenolic compounds may be negatively affected [16,17,18]. 

Enzymatic technologies have increased in popularity as greener methods for developing environmentally friendly processes. Enzymes enable reactions under moderate conditions, with higher selectivity and productivity. The potential uses of enzyme-assisted extractions include the solubilisation of fibre-bound phenolic compounds. The β-D-(1→4)-glucosidic and β-D-(1→4)-xylosidic bonds may be broken by the use of cellulolytic and xylanolytic enzymes, increasing the extractability of phenolics [19]. These enzymes have been applied to WB and OHs in order to improve the extracted compound content, which is an accessible and sustainable strategy compared with other methods with lower selectivity [20,21,22]. 

High hydrostatic pressure (HPP) is an emerging technology in the food industry that involves applying high pressure (100–600 MPa) to a product using liquid (usually water) for pressure transmission in a closed chamber [23]. The application of this technology implies a volume reduction during the pressure treatment, resulting in the modulation of chemical reactions. It is important to also highlight the potential impact of pressure on cells and tissues, damaging cell membranes, changing the conformation of membrane proteins, and partially or totally inactivating enzymes. HPP has been reported to reduce polyphenol oxidation-related enzyme activities, such as polyphenol oxidase and peroxidase activities [24,25], potentially enhancing the extractability of phenolic compounds. Although this technology has been conventionally used for food preservation, in recent years, HPP has also been identified as a promising strategy to improve the efficiency of bioactive compound extraction from different food matrices [26,27,28,29]. HPP application increases the bioaccessibility of antioxidant compounds and micronutrients, such as vitamins and minerals, thereby increasing the total antioxidant capacity of plant-based products [30,31]. The use of temperature combined with pressure can improve the efficiency of the outcome of the process.

Phenolic compounds are quite stable under pressure. Buckow et al. [32] reported that the use of high pressure prevented polyphenol degradation. Schilling et al. associated this behavior with the inactivation of certain enzymes, such as polyphenol oxidase, one of the enzymes involved in enzymatic polyphenol oxidation [33].

A combination of HPP and enzymatic hydrolysis treatments can be a strategy of interest in the development of more sustainable food ingredients with thermolabile bioactive compounds. These technologies enhance the extractability of bioactive compounds attached to the fibre. This strategy has been previously applied by different authors [34,35,36,37], although a combination of mild temperature conditions has scarcely been considered. 

Therefore, the main objective of this study is to determine the feasibility of using HPP at mild temperatures (40–70 °C) in combination with fibre-degrading enzymes as a strategy for increasing the antioxidant properties of WB- and OH-based ingredients.

## 2. Materials and Methods

### 2.1. Chemicals

Fluorescein, 6-hydroxy-2,5,7,8-tetramethyl-2-carboxylic acid (Trolox), 2,20-diazobis-(2-aminodinopropane)-dihydrochloride (AAPH), 2,2-diphenyl-1-picrylhydrazyl (DPPH), 2,20-azinobis 3-ethylbenzothiazoline-6-sulfonic acid (ABTS^•+^), Folin–Ciocalteu (FC) reagent, gallic acid (GA), iron (III) chloride hexahydrate (FeCl_3_∙6H_2_O), iron (II) sulfate heptahydrate (FeSO_4_∙7H_2_O), 2,4,6-tripyridyl-triazine (TPTZ), ferulic acid, hidroxybenzoic acid and p-coumaric acid were obtained from Sigma-Aldrich, Co. (St. Louis, MO, USA). Glucose oxidase-peroxidase (GOPOD) was supplied by Megazyme (Wicklow, Ireland). Sodium acetate, hydrochloric acid, glacial acetic acid and formic acid were purchased from PanReac AppliChem (ITW Reagents, Darmstadt, Germany). Different solvents were HPLC grade (Merck KGaA, Darmstadt, Germany, and Sigma Aldrich Co., Madrid, Spain). Viscoferm and UltraFlo XL food-grade enzymes were kindly supplied by Novozymes (Bagsværd, Copenhagen, Denmark).

### 2.2. Materials

WB was provided by Emilio Esteban, S.A. (Valladolid, Spain). Wheat (*Triticum aestivum* L.) was harvested in Valladolid in 2020. A dry milling process was used to separate the bran from the endosperm. OH was supplied by Sdad. Coop. Regional Ltda. Ribera del Duero (Burgos, Spain). Oat (*Avena sativa* L., var. Chimene) was harvested in Burgos during the 2019–2020 campaign. Chimene is a winter variety, has white grain and high productivity and is rich in protein. OH was separated from the grain using a mechanical dry system. Samples were milled and stored in vacuum plastic bags until further analysis. 

### 2.3. Nutritional Composition 

The nutritional composition of the raw ingredients, WB and OH, was determined. Dumas method, 990.03 [38], was used to determine the total protein content using an elemental analyser (LECO Corp., St. Joseph, MI, USA). To calculate the protein content, the conversion factors of 5.47 for wheat and 5.54 for oat were used. A petroleum ether extraction (40–60 °C) for 4 h in a Soxhlet extraction unit (AOAC 2005, method 2003.05) [38] was used to determine the total fat content. Moisture content was measured by drying three grams of powdered sample (WB, OH) at 105 °C for 3 h. For ash content, the samples were incinerated at 550 °C for 5 h in a muffle furnace (AOAC 2005, method 923.03) [38]. Carbohydrates were determined by difference. For total dietary fibre (TDF) content, the AOAC method 985.29 [38] was followed, with the use of the TDF100A-1KT test kit supplied by Sigma (St. Louis, MO, USA). K-PHYT, K-BGLU and K-TSTA-100A assay kits, provided by Megazyme (Wicklow, Ireland), were used to determine phytic acid (PA), β-glucan and total starch content (TSC), respectively. The results were expressed in g 100 g^−1^ of dry matter (d.m.). All analyses were performed in duplicate.

### 2.4. Processing Route

**Figure 1:**
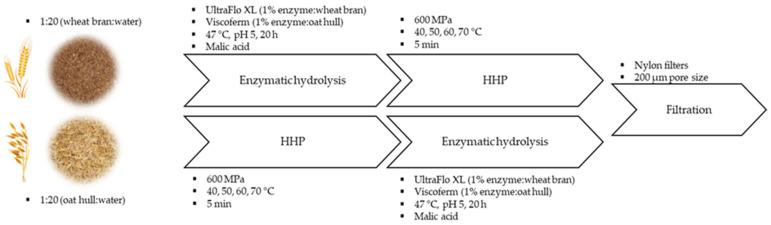
Processing route and operating conditions for the release of phenolic compounds, β-glucans and functionalisation of wheat bran (WB) and oat hull (OH).

Figure 1 shows a summary of the processing route and conditions applied to the samples. First, 100 g of each sample, WB and OH, were suspended in 2 L of distilled water (1:20, *w:v*). Subsequently, HPP treatment was evaluated at different temperatures before and after enzymatic hydrolysis.

For enzymatic hydrolysis, pH was adjusted to 5 using 1.5 M of malic acid, and 1% UltraFloXL or Viscoferm (enzyme to dry weight ratio, *w:w*) was added to the WB and OH suspension, respectively. The samples were incubated at 47 °C for 20 h. After this time, the samples were placed in a water bath at 95 °C for 10 min to inactivate the enzymes.

**Figure 2:**
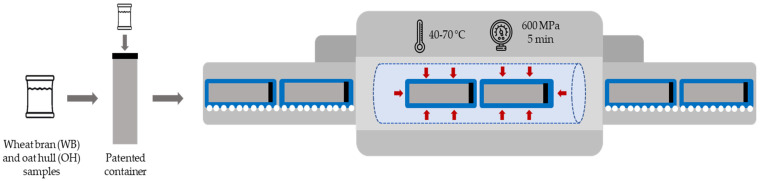
High-pressure process with container used to prevent heat exchange.

For HPP treatment, 4 different temperatures (40, 50, 60 and 70 °C) were applied with a pressure of 600 MPa for 5 min. Temperature was monitored over the entire cycle and the expected adiabatic increase was subtracted from the initial temperature in order to obtain the target temperature during treatment. For this purpose, the samples were placed in a patented container (WO 2017/031552) [39] with water at a specific temperature (Figure 2). The container was closed and placed inside the containers of the HPP equipment.

Finally, the samples were filtered using a nylon filter (200 μm mesh), aliquoted and stored at −20 °C until further analysis.

### 2.5. Total Phenolics (TP) Content 

Folin–Ciocalteu phenol reagent, according to the method described by Slinkard and Singleton [40], was used to determine the total phenol content (TPs). A gallic acid standard curve (98–700 μM) was prepared. Standards and sample absorbance were measured at 765 nm using a microplate reader (Fluostar Omega, BMG, Ortenberg, Germany). The results were expressed as μmol gallic acid equivalents (GAE) 100 g^−1^ d.m. All analyses were carried out in duplicate.

### 2.6. Determination of Phenolic Compounds by HPLC

Different samples were filtered with a 0.22 µm nylon filter. 

The different phenolic compounds were separated using an HPLC (Agilent 1200, Agilent Technologies, Santa Clara, CA, USA) with DAD (Agilent G1315B). The column used was Luna C18 (Phenomenex, Torrance, CA, USA), with a length of 250 mm, 2 mm internal diameter and 5 μm particle size. For gradient elution, milliQ water with 0.1% formic acid (solvent A) and acetonitrile with 0.1% formic acid (solvent B) were used. A volume of 20 μL of sample was injected. The working temperature was 25 °C and a flow rate of 0.4 mL/min with the following gradient was used: 0 min, 8% B; 10 min, 23% B; 15 min, 50% B; 20 min, 50% B; 23 min, 100% B. This was followed by a re-equilibration step. 

For quantification, authentic standard curves (gallic acid, hidroxybenzoic acid, ferulic acid and p-coumaric acid) were prepared in a range of concentrations between 0.1 and 25 μg mL^−1^, showing good linearity (R^2^ > 0.99). The results were expressed in mg 100 g^−1^ sample (d.m.).

### 2.7. Total Antioxidant Capacity (TAC)

For TAC determination, DPPH radical scavenging activity, oxygen radical absorbance capacity (ORAC), ABTS^•+^ radical cation scavenging activity and ferric-reducing antioxidant power (FRAP) assays were performed in duplicate.

#### 2.7.1. DPPH Radical Scavenging Activity

According to the method described by Brand-Williams et al. [41], the DPPH assay was performed with slight modifications. A 1:4:5 volume ratio of the sample, milliQ water and DPPH working solution (120 μM in pure methanol) were mixed and incubated for 30 min in darkness at room temperature. After incubation, the decrease in absorbance was measured on a microplate reader (Spectrostar Omega, BMG, Ortenberg, Germany). Trolox was used as a standard. The results were expressed as μmol of Trolox Equivalents (TE) 100 g^−1^ sample (d.m.).

#### 2.7.2. Oxygen Radical Absorbance Capacity (ORAC)

ORAC assay was performed following a method previously described by Ou et al. [42] with modifications. Phosphate buffer (75 mM, pH 7.4) was used to dilute the Trolox standard curve (7.5–210 μM) and samples. In a black 96-well microplate, 25 μL of sample, Trolox standard and phosphate buffer as blank were mixed with 125 μL of fluorescein and incubated at 37 °C for 3 min. Subsequently, 25 μL of AAPH solution was added to initiate the oxidation reaction, and fluorescence was monitored for 120 min with a microplate reader (CLARIOstar Plus, BMG, Ortenberg, Germany) using 485 nm excitation and 520 nm emission filters. To obtain the results, the area under the fluorescein decay curve was calculated as a function of Trolox concentration. Data were shown as μmol TE 100 g^−1^ sample (d.m.).

#### 2.7.3. ABTS^•+^ Radical Cation Scavenging Activity

ABTS^•+^ assay was performed in accordance with Re et al. [43], modified by Martin-Diana et al. [44]. A volume of 200 μL of ABTS^•+^ working solution was added to 20 μL of sample in a 96-well microplate. After 60 min, the decay in absorbance at 734 nm was recorded using a microplate reader (Spectrostar Omega, BMG Ortenberg, Germany). As a standard, a Trolox curve (7.5–210 μM) was prepared. The results were expressed as μmol TE 100 g^−1^ sample (d.m.).

#### 2.7.4. Ferric Reducing Antioxidant Power (FRAP)

FRAP was based on the method described by Benzie and Strain [45] with some modifications [9]. To prepare the FRAP working solution, acetate buffer (300 mM, pH 3.6), TPTZ solution (10 mM in 40 mM HCl) and FeCl_3_∙6H_2_O solution (20 mM) were mixed in a 10:1:1 volume ratio. A FeSO_4_∙7H_2_O curve (400–3000 μM) was prepared as a standard. In Eppendorf tubes, 20 μL of the sample and standard or distilled water as blank were mixed with 1.9 mL of FRAP working solution. The tubes were stirred and incubated for 5 min, and the absorbances were measured at 593 nm using a microplate reader (Spectrostar Omega, BMG Ortenberg, Germany). The results were expressed as mmol of Fe Equivalents (FeE) 100 g^−1^ sample (d.m.).

### 2.8. Statistical Analysis

The results were expressed as mean and standard deviation. Analysis of variance (ANOVA) and Duncan’s post hoc tests were carried out to detect differences between mean values. Statgraphics Centurion XVI^®^ software (StatPoint Technologies, Inc., Warrenton, VA, USA) was used to perform the statistical analyses. To elucidate the relationships between variables, a principal component analysis (PCA) was performed.

## 3. Results and Discussion

### 3.1. Nutritional Composition

The nutritional composition of the raw ingredients, WB and OH, was evaluated (Supplementary Table S1). The results showed that the protein content was 3.06 g 100 g^−1^ for OH, which was slightly lower than the values reported by He et al. [46] and Zhou et al. [47], who found values between 4.14 and 5.20 g 100 g^−1^, respectively. This difference could be attributed to the variety used in the study and agronomic conditions. In contrast, the protein content observed in WB was 15.16 g 100 g^−1^, within the range of previously reported values (13.19–15.97 g 100 g^−1^) [10,46]. 

Fat content was low in both samples (OH and WB), in accordance with previous studies [10]. The OH fat content was 0.61 g 100 g^−1^, which was lower than the results by Butt et al. and Bryngelsson et al. [48,49], who measured a fat content from 2 to 8.6 g 100 g^−1^. The WB fat content observed was 3.86 g 100 g^−1^, similar to values previously observed by the authors [50], but higher than those reported previously by other authors [51]. These differences in fat content could be due to the cultivar variety used or the method of production of the bran/hulls. 

Total carbohydrates were the main macronutrient in both ingredients, as shown in Supplementary Table S1. WB had a content of 74.73 g 100 g^−1^, values similar to those reported by different authors [10,52]. Meanwhile, in the case of OH, carbohydrate content was significantly higher compared to WB bran (92.04 g 100 g^−1^). 

Within the carbohydrate fraction, total dietary fibre (TDF) was also evaluated (Supplementary Table S1); DF content doubled in OH (90 g 100 g^−1^) compared to WB (45.24 g 100 g^−1^), resulting in agreement with the values previously reported by other authors [46,53]. The determination of TDF is an interesting parameter due to its relationship with bioactive properties since bound polyphenols are covalently linked to cell wall polysaccharides and frequently associated with diverse bioactive properties, such as anti-inflammatory, antioxidant or low glycemic index, among others [19,20]. Starch values were reported (Supplementary Table S1) as they can be an interesting parameter associated with the glycemic load of the final ingredient. Both raw materials, WB and OH, had low starch content (2.89 and 11.56 g 100 g^−1^ for OH and WB, respectively), in accordance with other authors since the endosperm contains most of the starch in the cereal grain [46].

The ash content (Supplementary Table S1) ranged between 4.30 g 100 g^−1^ and 6.26 g 100 g^−1^ for OH and WB, respectively, showing significant differences (*p* < 0.05) between the two ingredients. Similarly, the moisture content was significantly (*p* < 0.05) higher in WB than in OH, with values of 12.58 g 100 g^−1^ and 8.03 g 100 g^−1^, respectively. Ash and moisture content were similar to those previously reported by other authors [10].

Phytic acid (PA) is considered one of the most important anti-nutrients, interacting with minerals and reducing their solubility and absorption [54]. PA values were analysed for WB and OH, showing concentrations of 3.55 g 100 g^−1^ and 0.07 g 100 g^−1^, respectively. β-glucan content was determined, and this type of soluble fibre has demonstrated health-related benefits and may be affected by hydrolysis and the HPP process. The β-glucan values obtained were 0.07 g 100 g^−1^ in WB and 0.12 g 100 g^−1^ in OH, which were in the range of those reported by Dziki et al. [55]. 

**Figure 3:**
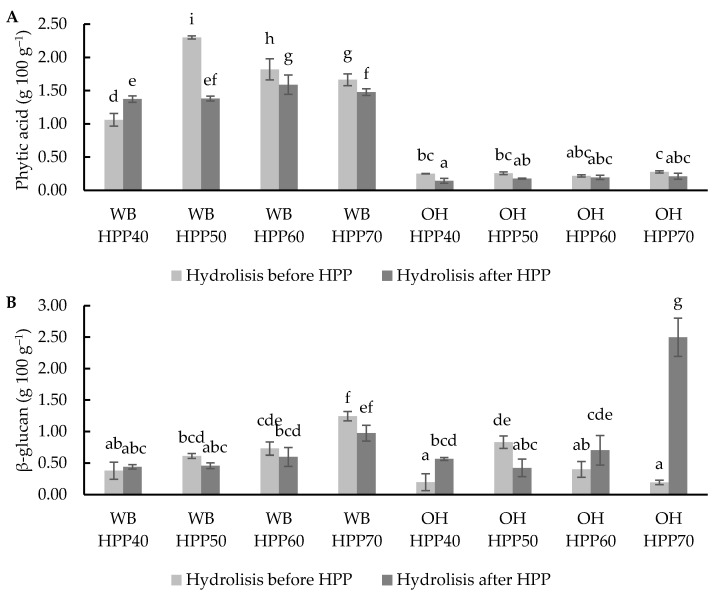
Phytic acid (**A**) and β-glucan (**B**) values in wheat bran (WB) and oat hull (OH) samples with hydrolysis before and after HPP at different temperatures. Results are expressed as g 100 g^−1^ of the sample. Different letters indicate significant differences (*p* < 0.05). Abbreviations: HPP: high-pressure processing.

HPP, temperature and enzymatic hydrolysis had significant effects on phytic acid and β-glucan concentration. The results agreed with the studies reported by other authors [56,57]. This effect can be applied as a strategy to tailor the final ingredient concentration, especially to reduce the content of PA and increase the values of β-glucan. The PA content of the hydrolysed and HPP-treated WB ranged from 1.06 g 100 g^−1^ to 2.3 g 100 g^−1^, with a significant (*p* < 0.05) effect of the temperature and the moment (before/after HPP) of enzymatic hydrolysis application (Figure 3A). The lowest levels of PA in WB were obtained when hydrolysis was applied before an HPP treatment at 40 °C. In the case of OH, PA values ranged between 0.14 g 100 g^−1^ and 0.28 g 100 g^−1^, with no significant differences due to different treatment temperatures. On the other hand, PA content was significantly (*p* < 0.05) higher in samples where HPP was applied before the hydrolysis. 

WB showed a significant increase in the β-glucan content after the treatment from 0.07 g 100 g^−1^ to 0.38 −1.24 g 100 g^−1^, and they were samples hydrolysed before HPP at 70 °C which showed the highest β-glucan content. Regarding OH, β-glucan values ranged from 0.19 to 2.5 g 100 g^−1^, with significant differences (*p* < 0.05) between treatments; the treatment at the highest temperature (70 °C) also resulted in the highest β-glucan content found when the hydrolytic treatment was applied after HPP. These values indicate that enzymatic hydrolysis combined with HPP enhances the release of β-glucan via the degradation of cellulose and hemicellulose, according to results reported in previous studies [58].

Due to the presence of native β-glucanase activity in these matrices, redox reactions or processing [59,60] β-glucan can be degraded into lower molecular weight (LMW) and oligosaccharide glucans, and glucose is not quantified according to the methodology used in this work. Associated to the degradation process it is expected that the presence of LMW β-glucans within the total beta-glucan quantified would affect their viscosity and hence their glucose and cholesterol absorption-lowering effects; on the other hand, interest in β-glucan degraded fractions (LMW and oligosaccharide) has been expressed from their antioxidant activities [61].

### 3.2. Total Phenolics (TPs)

**Figure 4:**
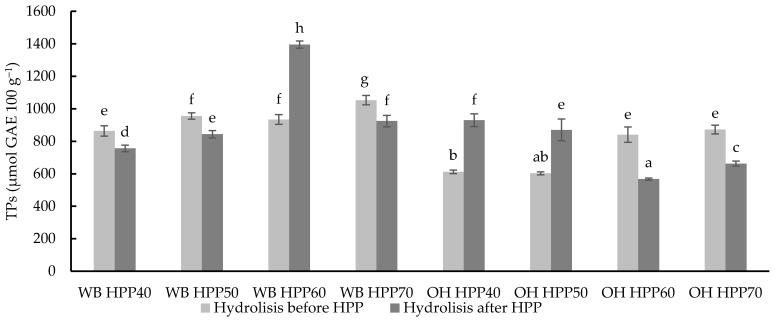
Total Phenolic compound (TP) content of different wheat bran (WB) and oat hull (OH) samples with hydrolysis before and after HPP at different temperatures. Results are expressed as µmol GAE 100 g^−1^ of dry matter. Different letters indicate significant differences (*p* < 0.05). Abbreviations: HPP: high-pressure processing.

Total Phenolic (TP) content was determined in the WB and OH samples hydrolysed before or after HPP treatment (Figure 4). According to Bautista-Expósito et al. [62], TP solubilisation depends on the type of enzyme and the conditions during the enzymatic treatment (enzyme/substrate ratio, temperature, pH, time, etc.). UltraFlo XL and Viscoferm at optimised conditions (pH 5, 47 °C for 20 h) were used to hydrolyse WB and OH, respectively, due to their higher performance as compared to other enzymes studied by the authors [22,62]. 

The application of HPP treatments has also been shown to improve the extraction of phenolic compounds [25]. 

The results showed significant differences (*p* < 0.05) in TPs between WB samples, which ranged between 756.47 and 1395.27 µmol GAE 100 g^−1^, values similar to other studies where the combination of temperature and HPP was used [51]. 

On the other hand, TPs in OH samples ranged from 566.91 to 930.45 µmol GAE 100 g^−1^, with significant differences (*p* < 0.05) between treatment conditions. The samples with the highest TP content were WB HPP60 with hydrolysis after the HPP treatment, followed by WB HPP70 with pre-hydrolysis, and OH HPP40 with hydrolysis following the HPP treatment, but with similar values to OH HPP60 and OH HPP70 with hydrolysis before HPP. Interestingly, an interactive effect of temperature and the moment of application of hydrolysis (before or after HPP) was observed in the case of OH, with higher TPs extracted from samples treated with lower HPP temperatures (40 and 50 °C) and hydrolysis after HPP, while hydrolysis before HPP was more efficient for higher TP content when HPP was carried out at higher temperatures (60 and 70 °C). This effect may be due to improved accessibility of enzymes to substrate after HPP treatment at mild temperatures (40–50 °C), while higher temperatures produce the contrary effect. On the other hand, similar TP levels with higher temperatures (60–70 °C) were extracted when the hydrolysis was already carried out before the HPP treatment; this may be explained by a synergistic effect between high pressure and hydrolysis, with its efficiency limited by temperature.

### 3.3. Determination of Phenolic Compounds by High-Performance Liquid Chromatography (HPLC)

HPLC was used to characterise and determine the main phenolic acids present in the WB and OH samples treated by enzymatic hydrolysis and HPP treatment. Table 1 shows the quantification of major phenolic compounds in the WB samples. It was observed that higher temperatures increased the extractability of phenolic compounds. The application of hydrolysis before HPP treatment increased the content of gallic and hydroxybenzoic acids, up to 33.35 and 2.12 mg 100 g^−1^ for gallic acid and hydroxybenzoic acid, respectively. This effect was also observed in the case of ferulic acid, the phenolic acid most abundant in cereals [10,63], except for samples treated at 70 °C. Ferulic acid content was higher in samples treated with HPP after the hydrolysis process, although the highest value was observed in samples treated with hydrolysis and HPP at 70 °C, reaching 348.41 mg 100 g^−1^.

The results of phenolic acids determined in OH samples are shown in Table 2. In this case, the HPP process with moderate temperatures (40, 50 °C) resulted in the highest content of phenolic compounds. Hydroxybenzoic acid concentration ranged from 1.13 to 4.21 mg 100 g^−1^, *p*-coumaric acid from 0.17 to 0.42 mg 100 g^−1^ and ferulic acid from 1.03 to 5.61 mg 100 g^−1^; the concentration of these compounds was similar to that reported in previous studies [64], where increased solubilities of ferulic and *p*-coumaric acids via enzymatic hydrolysis were reported [22]. The sample with the highest phenolic compound content was OH HPP40 + H.

### 3.4. Total Antioxidant Capacity (TAC)

The total antioxidant capacity (TAC) of the samples was determined by four different methods: 2,2-diphenyl-l-picrylhydrazyl assay (DPPH), oxygen radical absorbance capacity (ORAC), 2,20-azino-bis (3-ethylbenzothiazoline-6-sulfonic acid) (ABTS^•+^) and ferric-reducing ability assay (FRAP).

**Figure 5:**
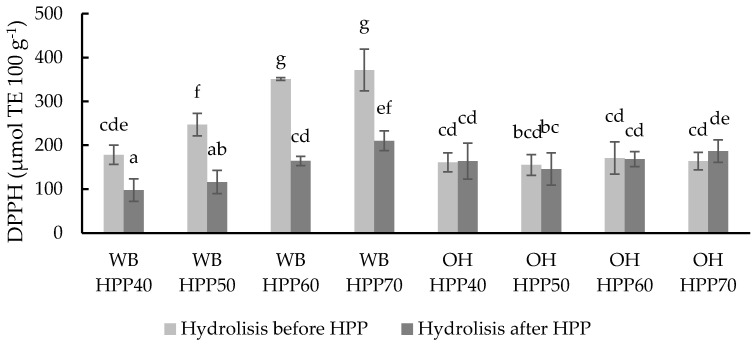
DPPH values of different wheat bran (WB) and oat hull (OH) samples with hydrolysis before and after HPP at different temperatures. Results are expressed µmol TE 100 g^−1^ of the sample. Different letters indicate significant differences (*p* < 0.05). Abbreviations: HPP: high-pressure processing.

Anti-radical activity against DPPH of the different samples was measured (Figure 5). WB samples had values between 97.78 and 371.47 µmol eq. Trolox 100 g^−1^ showed significant differences (*p* < 0.05) between treatment conditions. Increasing temperatures resulted in increasing the TAC of WB samples, regardless of the application of hydrolysis before or after the HPP treatment, although the application of hydrolysis before HPP in WB resulted in significantly higher DPPH values. WB HPP70 was the sample with the highest antioxidant capacity against the DPPH radical. On the other hand, OH sample values were not significantly affected by the different treatment parameters, ranging from 145.70 to 186.62 µmol eq. Trolox 100 g^−1^.

This assay allows the evaluation of the antioxidant capacity, which, despite not being a natural radical, reacts in a similar way to the neutralisation reactions of peroxyl radicals [65]. Therefore, phenolic compounds found in samples can potentially be involved in preventing peroxyl-mediated reactions, such as lipid oxidation [66].

The increase in DPPH antiradical activity can be associated with an increase in the phenolic content, as observed from the correlation between the TP and DPPH results. Previous studies [34] showed the enhancement in the extractability of phenolic compounds after the use of HHP. The temperature levels used (below 70 °C) did not affect the bioactivity of the resulting ingredients negatively. On the contrary, increasing temperature when treating WB resulted in increasing DPPH values. This last effect was not observed in the case of OH. Previous work showed improvements in the total phenolic content of wheat and oat bran (22.49% and 25.84%, respectively) and DPPH activity (24.41% and 15.88%, respectively) when 80 °C ultrasound-assisted thermal treatment was used, indicating that the thermal stability observed in the present work is in accordance with the results from Călinoiu and Vodnar [67]. 

The antioxidant activity was heightened when pressure was applied after hydrolysis, which points out the possibility that the decompression at the end of the HPP treatment has a decisive role in the breakdown of the cell wall due to the fast gas expansion dissolved through pressurisation [68], an effect that was significant in the cell wall degradation achieved by the hydrolytic enzymes.

**Figure 6:**
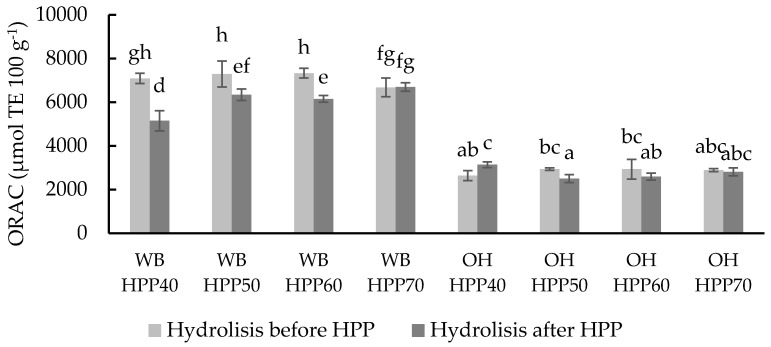
ORAC values of different wheat bran (WB) and oat hull (OH) samples with hydrolysis before and after HPP at different temperatures. Results are expressed µmol TE 100 g^−1^ of the sample. Different letters indicate significant differences (*p* < 0.05). Abbreviations: HPP: high-pressure processing.

Oxygen radical absorbance capacity (ORAC) was also determined to evaluate the peroxyl scavenging capacity through hydrogen atom transfer [69]. Similarly to the previous radical assay (DPPH), carrying out the hydrolysis before the HPP treatment resulted in higher TAC values for WB (Figure 6). The values ranged from 5145.20 to 7326.35 µmol eq. Trolox 100 g^−1^ in WB samples, and from 2500.61 to 3135.63 µmol eq. Trolox 100 g^−1^ in OH samples, with significant (*p* < 0.05) increases in ORAC due to temperature increase in WB samples hydrolysed after the HPP treatment.

**Figure 7:**
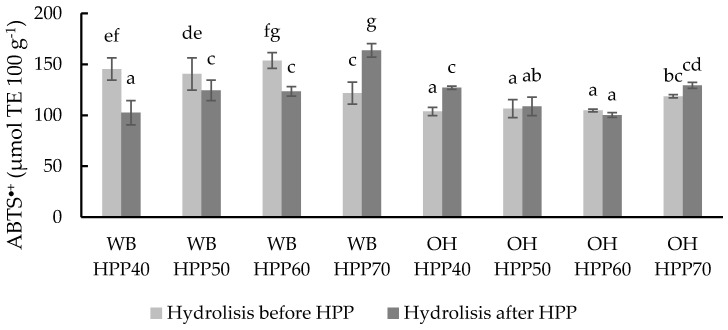
ABTS values of different wheat bran (WB) and oat hull (OH) samples with hydrolysis before and after HPP at different temperatures. Results are expressed µmol TE 100 g^−1^ of the sample. Different letters indicate significant differences (*p* < 0.05). Abbreviations: HPP: high-pressure processing.

The different samples were also evaluated against the ABTS^•+^ radical (Figure 7). This assay evaluates the electron transfer reactions of the antioxidants similarly to the DPPH assay [70]. The results obtained showed significant differences (*p* < 0.05) between some of the treatment factors, ranging from 102.50 to 163.81 µmol eq. Trolox 100 g^−1^ for WB samples and from 100.19 to 129.42 µmol eq. Trolox 100 g^−1^ for OH samples. Similarly to the ORAC results, a positive correlation between the temperature of the HPP treatment and ABTS^•+^ values was observed in the case of WB hydrolysed after the HPP treatment. The highest ABTS^•+^ values were WB HPP60 and OH HPP70, with hydrolysis before and after the HPP treatment, respectively.

**Figure 8:**
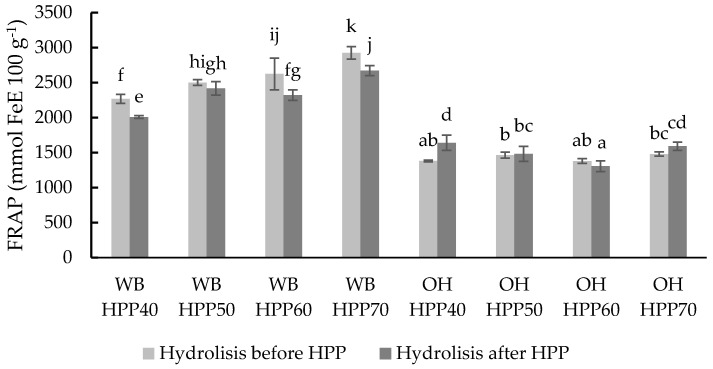
FRAP values of different wheat bran (WB) and oat hull (OH) samples with hydrolysis before and after HPP at different temperatures. Results are expressed mmol FeE 100 g^−1^ 100 g ^−1^ of the sample. Different letters indicate significant differences (*p* < 0.05). Abbreviations: HPP: high-pressure processing.

The reducing power of the hydrolysed and HPP-treated WB and OH samples before and after hydrolysis was determined using the FRAP assay (Figure 8). The samples showed similar behaviour as observed with DPPH results in terms of temperature and hydrolysis application (before or after HPP).

FRAP values ranged from 2006.59 to 2924.76 mmol eq. Fe 100 g^−1^ in the WB samples and from 1305.23 to 1640.47 mmol eq Fe 100 g^−1^ in the OH samples. A correlation between the temperature and FRAP value in the WB samples was observed, with increasing temperatures resulting in increasing reducing potential, both in samples hydrolysed before and after HPP, with higher values in the first case.

**Figure 9:**
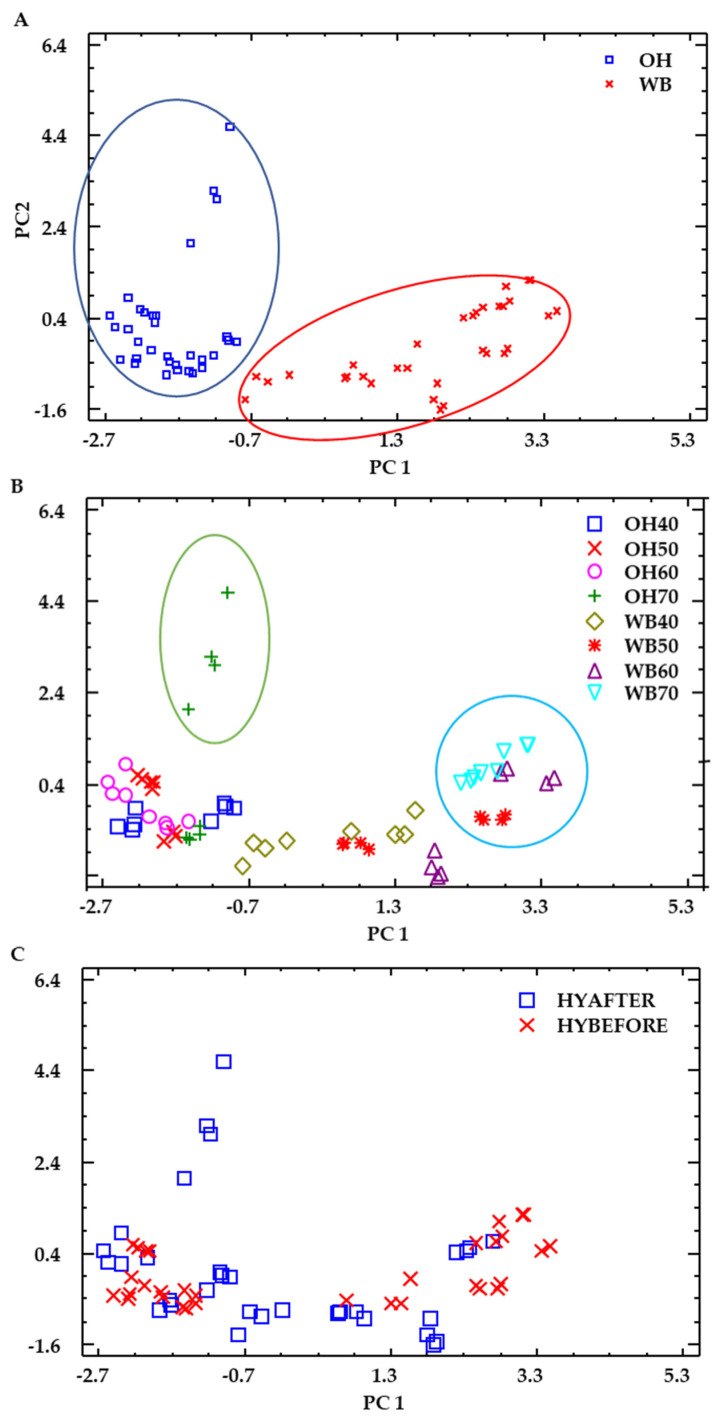
PCA analysis. (**A**) Labels: raw material, WB and OH. (**B**) Labels: raw matter and HPP temperature. (**C**) Labels: hydrolysis application (before or after HPP).

**Figure 10:**
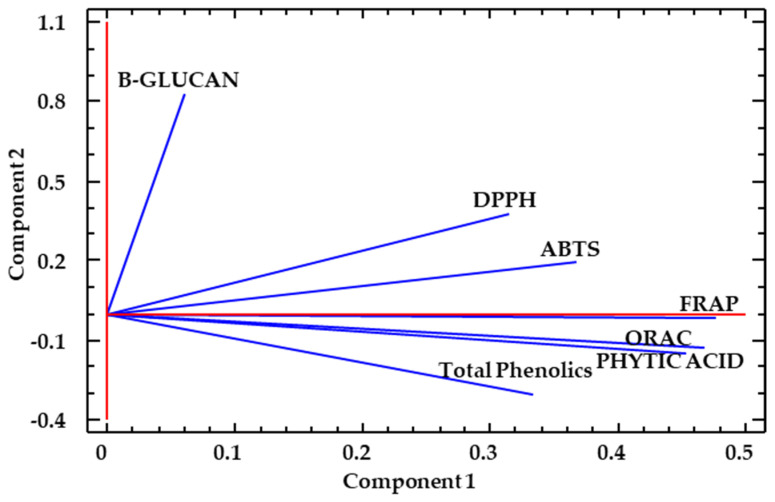
PCA analysis. Contribution of the different bioactivity markers analysed: Beta-glucan, phytic acid, total phenolics and total antioxidant parameters (DPPH, ABTS, ORAC and FRAP) for wheat bran (WB) and oat hull (OH) treated with HPP at different temperatures (40, 50, 60 and 70 °C) before or after hydrolysis.

A principal component analysis was carried out to illustrate the variation in phytic acid, β-glucan and bioactivity of WB and OH samples, and to identify correlations of variables (Figure 9 and Figure 10). The variance of the data explained by the first and second principal components (PC 1 and PC 2) was 58.6 and 17.4, respectively, with a cumulative percentage of 76,0. The distribution of WB samples (Figure 9A) was better explained by component 1 (PC 1), a component with important participation of phytic acid, TPs (where ferulic acid was majoritarian) and antioxidant markers (Figure 10). OH samples, on the other hand, were better explained by PC 2, which was highly influenced by the β-glucan content. The significant effect of temperature observed in some of the bioactive compounds and antioxidant determinations can be reflected in the distribution shown in Figure 9B, where circled samples correspond to the higher temperature treatments. In Figure 9C, the distribution according to the application of the hydrolysis treatment before or after HPP can be observed; in the case of WB samples (right side, as shown in Figure 9A), the samples corresponding to hydrolysis before HPP were distributed more abundantly in the right part of the PC 1, which was positively contributed by the antioxidant parameters (Figure 10).

## 4. Conclusions

These findings support the utilisation of cereal processing industry byproducts as bioactive compound sources through the combined HPP/thermal/hydrolysis processing and transforming them into valuable functional ingredients for the food industry. To the best of our knowledge, this is the first study to investigate this strategy in wheat bran and oat hull.

The combination of the two processes, enzymatic hydrolysis and high-pressure thermal processing (HPP), produced a significant increase in phenolic compounds in WB and OH, especially in ferulic acid, which has been previously associated with the increased antioxidant capacity of cereal bran. In addition, these processes reduced the content of anti-nutrient phytic acid in WB samples, which interfered with the absorption of minerals such as calcium, iron and zinc. 

High temperatures (60–70 °C) during the HPP process favoured the release of phenolic compounds linked to fibre, especially ferulic and gallic acid in WB and ferulic and hydroxybenzoic acid in OH, as well as a higher extractability of β-glucan, increasing up to 19 and 21 times in WB and OH, respectively. Enzymatic hydrolysis was more effective when performed before the HPP treatment in most of the cases. This is an interesting outcome from these processing conditions, as HPP after hydrolysis would significantly increase the product stability and shelf life since it is a pasteurisation process. Phenolic compounds and β-glucans are involved in many bioactive properties, providing health benefits after their consumption. One of the most important compounds identified in WB is ferulic acid; different studies suggest that it possesses anti-inflammatory, anti-diabetic, anticancer, and cardioprotective properties. β-glucan promotes the growth and development of health-friendly intestinal microflora and enhances the antioxidant properties of products in which it is formulated [71,72,73,74]. Limitations of these results can be associated with the variability of the sources, for instance. Different studies have shown a wide protein or phenolic range variability in cereal byproducts associated with cultivar varieties and the environmental conditions of growing [9,67].

This study shows that the application of combined strategies (hydrolysis and HPP) favour obtaining new functional ingredients, with a higher content of phenolic compounds and β-glucans, as well as increased antioxidant activity. The proposed processing enables the derived cereal byproducts bran/hull to be perceived as significant valuable sources for the food industry, increasing their value through the recovery of bioactive compounds suitable for serving as streams for their formulation into nutraceuticals and functional foods and reducing the environmental impact.

## Figures and Tables

**Table 1:** Phenolic compounds quantified by HPLC in wheat bran (WB) samples with hydrolysis before and after HPP at different temperatures. Results are expressed in mg 100 g^−1^ of the sample. Different letters indicate significant differences (*p* < 0.05).

	Gallic Acid	Hidroxybenzoic Acid	p-Coumaric Acid	Ferulic Acid
WB H + HPP 40	n.d.	0.60 ± 0.02 ^a^	<LOD	21.61 ± 1.53 ^a^
WB H + HPP 50	12.49 ± 0.11 ^a^	0.56 ± 0.05 ^a^	<LOD	209.81 ± 1.90 ^c^
WB H + HPP 60	17.42 ± 0.00 ^b^	0.64 ± 0.02 ^ab^	<LOD	191.17 ± 0.80 ^b^
WB H + HPP 70	n.d.	0.70 ± 0.05 ^b^	<LOD	348.41 ± 5.54 ^f^
WB HPP 40 + H	24.22 ± 0.51 ^c^	1.49 ± 0.04 ^c^	<LOD	315.54 ± 10.47 ^e^
WB HPP 50 + H	33.35 ± 4.42 ^d^	1.49 ± 0.04 ^c^	<LOD	234.37 ± 1.60 ^d^
WB HPP 60 + H	22.47 ± 0.88 ^c^	2.03 ± 0.07 ^d^	<LOD	228.86 ± 0.47 ^d^
WB HPP 70 + H	13.03 ± 0.11 ^a^	2.12 ± 0.03 ^d^	<LOD	213.69 ± 3.24 ^c^

Abbreviations: HPP: high-pressure processing, n.d.: not detected, LOD: limit of detection.

**Table 2:** Phenolic compounds quantified by HPLC in oat hull (OH) samples with hydrolysis before and after HPP at different temperatures. Results are expressed as mg 100 g^−1^ of the sample. Different letters indicate significant differences (*p* < 0.05).

	Gallic Acid	Hidroxybenzoic Acid	p-Coumaric Acid	Ferulic Acid
OH H + HPP 40	n.d.	2.38 ± 0.04 ^b^	<LOD	2.53 ± 0.01 ^c^
OH H + HPP 50	n.d.	3.18 ± 0.01 ^d^	<LOD	4.69 ± 0.07 ^f^
OH H + HPP 60	n.d.	2.41 ± 0.02 ^b^	<LOD	2.68 ± 0.06 ^cd^
OH H + HPP 70	n.d.	1.13 ± 0.01 ^a^	<LOD	1.03 ± 0.10 ^a^
OH HPP 40 + H	n.d.	4.21 ± 0.06 ^g^	0.42 ± 0.00 ^c^	5.61 ± 0.08 ^g^
OH HPP 50 + H	n.d.	2.72 ± 0.00 ^c^	0.17 ± 0.01 ^a^	2.76 ± 0.12 ^d^
OH HPP 60 + H	n.d.	3.29 ± 0.02 ^e^	0.25 ± 0.16 ^ab^	4.08 ± 0.07 ^e^
OH HPP 70 + H	n.d.	3.50 ± 0.02 ^f^	0.36 ± 0.03 ^bc^	1.44 ± 0.03 ^b^

Abbreviations: HPP: high-pressure processing; n.d.: not detected; LOD: limit of detection.

## Data Availability

Data is contained within the article or supplementary material.

## Supplementary Materials

The following supporting information can be downloaded at: https://www.mdpi.com/article/10.3390/foods13030378/s1. Table S1. Nutritional composition of wheat bran (WB) and oat hull (OH) samples.
