# Supplementary material for: Combined Strategy Using High Hydrostatic Pressure, Temperature and Enzymatic Hydrolysis for Development of Fibre-Rich Ingredients from Oat and Wheat By-Products
Source: Foods. 2024 Jan 24;13(3):378. doi: 10.3390/foods13030378 (PMC10855855; doi:10.3390/foods13030378)
Supplement: Supplementary file 1 [file foods-13-00378-s001.zip › foods-2821433-supplementary.pdf]

Supplementary Supplementary Table S1. Nutritional composition of wheat bran (WB) and oat hull (OH) samples. Values were expressed as g 100 g^{-1} of dry matter. Abbreviations: TDF: total dietary fibre, PA: phytic acid. Different letters indicate significant differences ($p < 0.05$).

	WB	OH
**Protein**	15.16 ± 0.40 [b]	3.06 ± 0.07 [a]
**Fat**	3.86 ± 0.20 [b]	0.61 ± 0.01 [a]
**Carbohydrates**	74.73 ± 0.12 [a]	92.04 ± 0.07 [b]
**TDF**	45.24 ± 13.93 [a]	89.64 ± 0.10 [b]
**Starch**	11.56 ± 0.83 [b]	2.89 ± 0.48 [a]
**Ash**	6.26 ± 0.08 [b]	4.30 ± 0.00 [a]
**Moisture**	12.58 ± 0.61 [b]	8.03 ± 0.02 [a]
**PA**	3.55 ± 0.02 [b]	0.07 ± 0.00 [a]
**β-glucan**	0.07 ± 0.01 [a]	0.12 ± 0.03 [b]
